# Supplementary material for: Radio-transparent dipole antenna based on a metasurface cloak
Source: Nat Commun. 2022 Mar 2;13:1114. doi: 10.1038/s41467-022-28714-w (PMC8891352; doi:10.1038/s41467-022-28714-w)
Supplement: Supplementary file 1 — Supplementary Materials [file 41467_2022_28714_MOESM1_ESM.pdf]

Supplementary Information for

Radio-Transparent Dipole Antenna Based on a Metasurface Cloak

Jason Soric[1,§], Younes Ra'di[2,§], Diego Farfan[1,2], and Andrea Alù[1,2,3,4,*]

[1]*Department of Electrical and Computer Engineering, The University of Texas at Austin, Austin, TX 78712, USA*

[2]*Photonics Initiative, Advanced Science Research Center, City University of New York, NY 10031, USA*

[3]*Physics Program, Graduate Center, City University of New York, NY 10016, USA*

[4]*Department of Electrical Engineering, City College of The City University of New York, NY 10031, USA*

[§]*These authors contributed equally to this work.*

[*]*To whom correspondence should be addressed:* aalu@gc.cuny.edu

Supplementary Note 1. Scattering from a metasurface integrated within a dielectric rod for improved angular stability and bandwidth suppression

The inset of Supplementary Fig. 1 illustrates the cross-section of the cylindrical target under study. Here region 1 is the central region for $\rho < r_1$, region 2 is for $r_1 < \rho < r_2$, region 3 is for $r_2 < \rho < r_3$, and region 4 is free space for $\rho > r_3$. While the model is completely general, we only consider non-magnetic materials, such that, $\mu_l = \mu_0$, as they introduce unwanted passive intermodulation in high power sources such as base stations. It is interesting to first consider the required surface impedance across a wide frequency band for solid core dielectric rods of two readily available materials with a conformal cover at normal incidence to appreciate the required surface impedance with geometry and material composition. In Supplementary Fig. 1, we increase the diameter of each rod to consider the required surface impedance to minimize the normalized total scattering

width (SW), defined as $\sigma_{2D}/\lambda = \frac{2}{\pi \sin\theta_i} \sum_{n=0}^{N_{\max}} (2-\delta_{0n}) \left| c_n^{TM} \right|^2$ where δ_{0n} is the Kronecker discrete delta function, λ is the free-space wavelength, and $N_{\max}$ is the maximum relevant order. We safely consider the ultrathin conductive impedance layer as being lossless and isotropic, such that, $Z_{zz} = jX_s$. Supplementary Fig. 1 illustrates two important features of the choice of realizable scalar impedance surfaces. Electrically small targets of low dielectric composition have strong frequency dispersion across the band and also require large effective surface impedances. Therefore, in a practical design for large bandwidth performance, dielectric targets of moderate electrical cross-section should be considered. Care must also be taken to not introduce TE-polarized scattering by choosing too large the electrical cross-section, which in most designs can be 20 dB lower than that of the dominant TM-polarized wavefronts for the frequencies considered here. Said explicitly, we do not aim to reduce both polarizations, but significantly reduce the dominant mode, while not enhancing the already much lower higher-order modes.

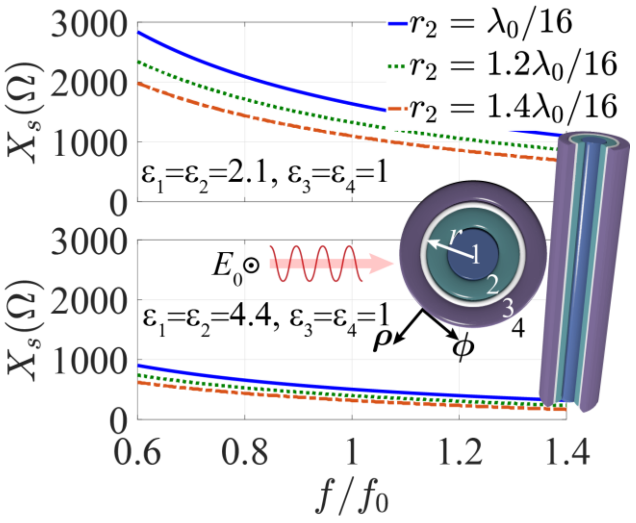

Supplementary Figure 1. Required surface impedance values (X_s) from the minimization of scattering width (SW) at normal incidence for typical dielectrics with increasing cross-section dimensions.

Our approach is based on the reduction of scattering from moderate-valued dielectric rods of low electrical cross-section [S1] . By choosing this route, we leverage several features previously unexplored. The scattering of dielectric rods is already much less than comparable conductive targets at all angles and polarizations; therefore, simple scalar covers targeting the dominant TM polarization are appropriate. Additionally, conformal covers optimize the bandwidth for dielectric targets, which is diametric to the bulky covers targeting conductive targets [S2] -[S3] , or with high dielectric substrates [S4] . The simplest inductive surface for dominant TM polarization is that of traces aligned with the incident wavefront [S5]

$$Z_{strips}^{TM} = j\omega \frac{\mu_0 D}{2\pi} \ln\left[\csc\left(\frac{\pi w}{2D}\right)\right]\left(1 - \frac{\cos^2\theta_i}{2\varepsilon_{eff}}\right). \quad (1)$$

This simple but accurate formula calculates the effective shunt inductance for a given infinite planar interface, with trace width w, period D, and $\varepsilon_{eff} = (\varepsilon_l + \varepsilon_{l-1})/2\varepsilon_0$. It is well-known that thin angular stable surfaces for various applications are difficult to design while maintaining their functionality [S5] -[S7] , and this intrinsic angular sensitivity is clearly seen in (1) as well. Different from cloaking applications in the strict sense [S1] , here we immerse the inductive screen inside the target rather than covering it to improve the angular stability and suppression bandwidth. In Supplementary Fig. 2, we demonstrate the improvements in angular stability and bandwidth across a large frequency band. The analytical SW in Supplementary Fig. 2 includes the elevation angular dependence for realizable simple covers as well as their frequency dispersion using (1). We emphasize two important considerations here. First, this model does not include polarization coupling effects, due to the anisotropy of realistic covers, which may affect the angular performance. Second, these analytical results are for an infinitely long rod, which does not include

longitudinal resonances, end effects, and relevant to this work, the input impedance at the feedgap when formed into an antenna. Still, these analytical results give us great insight into the effect of surface immersion suggested in this work.

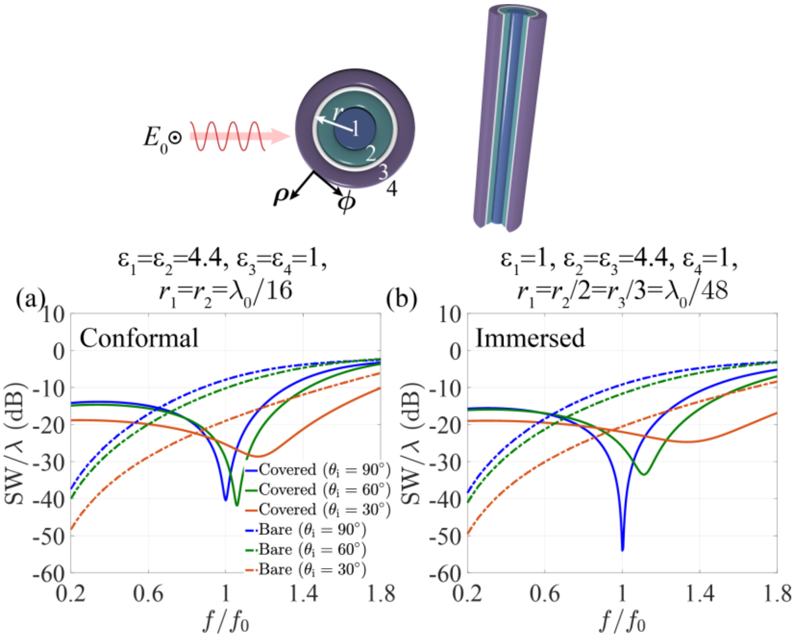

Supplementary Figure 2. Scattering width (SW) with incident angle for conformal and immersed covers. E_0 is the incident electric field.

Supplementary Fig. 2 considers the angular SW response across the band of interest for different incident angles. Here, we choose the same surface impedance calculated by (1)-(2) of the main paper for a given frequency at normal incidence and then use (1) to take into account frequency dispersion and angular dependence of a practical impedance surface. For the conformal design, the dielectric rod is solid with $\varepsilon_l = \varepsilon_c = 4.4$ and $d = 21.9mm$. A surface impedance of $j1.34\eta_0$ (where η_0 is free space wave impedance) is applied to the surface, as calculated for normal incidence for

$f/f_0 = 1$. As it can be seen from the figure, there is a blue shift in the minimum scattering dip as the incident angle increases which is fully consistent with the experimental work in Ref. [S8] .

Next, we consider an immersed mantle cloak design. The total width of both designs is the same. For the immersed case, the dielectric is inhomogeneous, where the center has been hollowed out for antenna mounting and weight reduction, as well as capacitive feeding for passive intermodulation reduction. The improvement in angular stability and suppression bandwidth is significant in the immersed case. As well, the overall visibility of the sample is now much lower across the entire band, consistently being better than that of the conformal design. Now the required surface impedance is $j1.1\eta_0$ at $f/f_0 = 1$ for normal incidence.

Supplementary Note 2. Dual-polarized spectral and angular response for the designed Radio-Transparent Dipole Antenna Based on a Metasurface Cloak (RTDA)

To verify the angular response across a large bandwidth, we excite the RTDA designed for Fig. 3 of the main text with dual-polarized plane waves along the elevation plane. The optimized antenna has a trace width of w with a period $D = a_2 / \pi$. Before we proceed with explaining the simulation results, it should be noted that, although, we are only using two strips, effectively, the structure is a periodic metasurface with periodicity of $0.28\lambda_0$. Based on our theoretical analysis (based on satisfying boundary conditions), the required impedance for scattering cancellation at 3.5 GHz is $j243.6$ Ohms. We plug in this required impedance value into Eq. 3 and find the periodicity and width of the strips. Based on the analytical formula given in Eq. 3 of the paper, the vertical strips metasurface used in Fig. 3 of the paper should provide an impedance of $j249.8$ Ohms at the design frequency which is very close to the required value. To verify that the impedance surface provides

the required value, we calculated, in full-wave simulation, the surface impedance of the unwrapped metasurface, i.e., periodic array of infinitely long strips with the same width and periodicity that we have used in our design. The right panel in Supplementary Fig. 3a shows the real and imaginary parts of surface impedance from full-wave simulations. It can be seen that at the design frequency (3.5 GHz), the array provides $0.27 + j240.52\,\text{Ohm}$ that is very close to what is required. In Supplementary Fig. 3, we still demonstrate that this design gives very good bistatic scattering suppression, and more impressively, with an angular stable response over a very broad bandwidth. One obvious discrepancy, is now the TE-polarized wavefronts introduce scattering beyond that of the conductive dipole for oblique incidence. While this is important for dual-polarized antennas, there are two main consequences of this. First, this TE scattering enhancement simply limits the bandwidth for dual-polarized sources; however, the bandwidth is still remarkable and well-above most other cloaking strategies. Second, this simply implies this particular design is best suited for moderate to high gain systems. For the system considered in this work, the gain of the reflector-backed array is high enough to not be drastically affected by large off-angle radiation. We note that the elevation angle in this study has been redefined to consider the beamwidth of incident radiation [see Supplementary Fig. 3a]. Considering the specification of both the LB and HB BW to be < 70 deg., we expect significant radiation for $60^\circ < \theta_i < 120^\circ$.

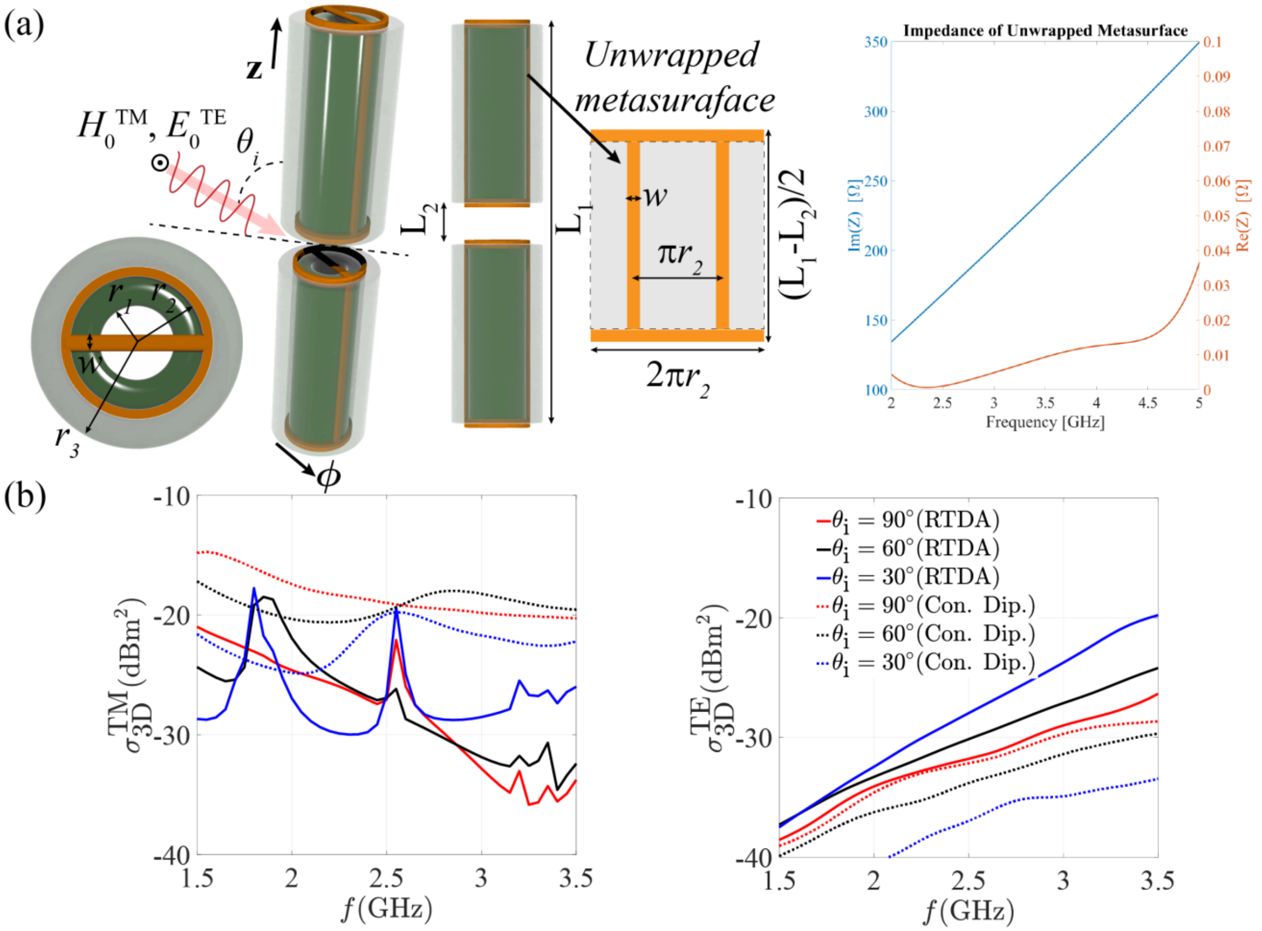

Supplementary Figure 3. (a) Schematic of the Radio-Transparent Dipole Antenna Based on a Metasurface Cloak (RTDA). Design parameters: $L_1 = 231\text{mm}$, $L_2 = 36.4\text{mm}$, $r_1 = 4.77\text{mm}$, $r_2 = 7.77\text{mm}$, $r_3 = 10.77\text{mm}$, and $w = 1.5\text{mm}$. These dimensions are the same for the design in Fig. 3 of the main text. The right panel shows the real and imaginary parts of surface impedance (from full-wave simulations) of the unwrapped metasurface, i.e., periodic array of infinitely long strips with the same width and periodicity that we have used in our design. It can be seen that at the design frequency (3.5 GHz), the array provides $0.27 + j240.52\,\text{Ohm}$ that is very close to what is required ($j243.6$ Ohms). Here, H_0^{TM} and E_0^{TE} are the magnetic field of the incident transverse-magnetic-polarized wave and the electric field of the incident transverse-electric-polarized wave,

respectively. (b) Dual-polarized spectral and angular response for the designed RTDA. Simulations have been performed using CST STUDIO SUITE [S9] .

We note several narrowband scattering peaks across the band at 1.74, 2.5, and 3.25 GHz. These effects are caused by the realistic anisotropy of the cover and longitudinal resonances of the finite length antenna. Again, this points to the necessity of using a simplified surface for broadband applications with angular rich near-field sources.

Supplementary Note 3. Design of a simple cloak for practical antenna applications

As discussed previously, large bistatic suppression bandwidths and angular spectra are already difficult design challenges in their own right. An equally important consideration is that the low-observable dipole arms, when formed into an antenna, radiate with industry-level standards. These lowband (LB) specifications require that the antenna have a return loss better than 10 dB when matched to a standard 50 Ω system; the 3 dB beamwidth $(\mathrm{BW}) < 70^{\circ}$, and beam squint $< \pm 7^{\circ}$, thus, directing base station links efficiently into predefined sectors.

To demonstrate the improvements offered by our design, we test this proposed cloaking method with a unit cell of a real basestation antenna panel. In Supplementary Fig. 4, we show the geometry of this single unit cell. Placed about a quarter of the central wavelength (at 2.2 GHz) above the panel backplane are six dual-polarized highband (HB) elements. A large LB cross-dipole is then placed about a quarter of its central wavelength (at 833 MHz) above the same backplane. Due to the close proximity of the HB and LB elements, a very low-profile and conformal design is needed,

which is difficult to obtain with bulky covers designed to reduce the scattering of conductive targets over a large bandwidth in conventional approaches, in this case, a conventional cylindrical dipole [S2] -[S3] . The design presented here is much easier to match across a large bandwidth by the simple usage of a stripline matching network to a 50 Ohm standard port (Supplementary Fig. 4d), using a planar stub and a $\lambda/4$ transformer, implemented on a 3-layer board as is typically done in such antenna systems. Fine-tuning of the matching and beamwidth was also made by adjusting the feedgap and distance to the reflector. Detailed design dimensions are given in Supplementary Figs. 4b, 4c, and 4d. The fabricated panel is shown in Supplementary Fig. 4e.

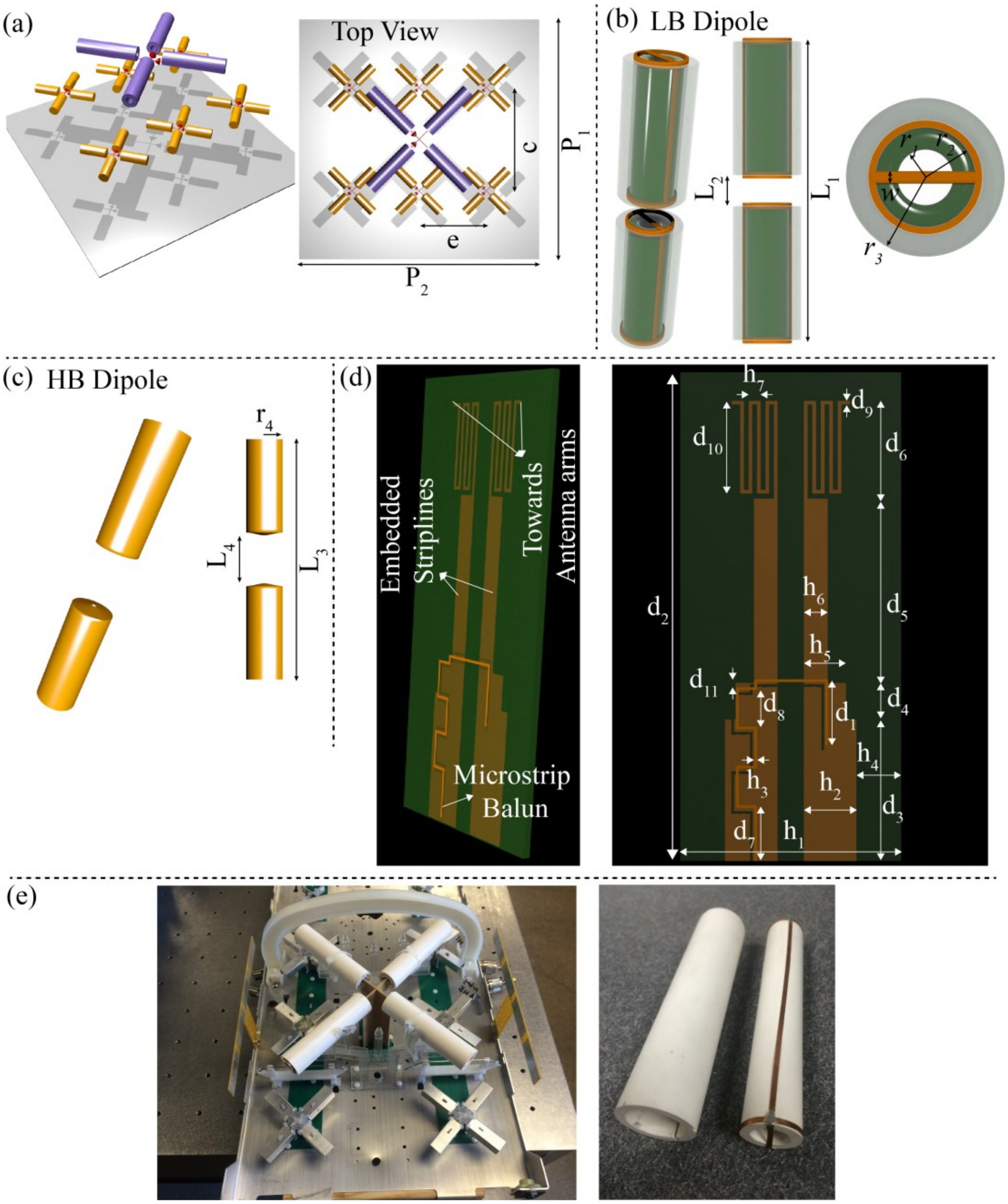

Supplementary Figure 4. (a) Geometry of the single panel. (b) A lower band (LB) radio-transparent dipole antenna based on a metasurface cloak (RTDA) arm. (c) A higher band (HB) RTDA arm. (d) Differential stripline matching network for the RTDA: the circuit is composed of to striplines embedded in a FR4 dielectric with thickness of 1.57mm. The striolines are located right at the middle of the dielectric. A microstrip balun is used to feed the striplines. The striplines are directly connected to the arms of the cloaked antenna at their ends. Note that similar matching

network is used for both of the orthogonally located dipoles. (e) Fabricated panel. Design parameters: $P_1 = P_2 = 352\text{mm}$, $e = 112\text{mm}$, $c = 172\text{mm}$, $L_1 = 231\text{mm}$, $L_2 = 36.4\text{mm}$, $L_3 = 100\text{mm}$, $L_4 = 18\text{mm}$, $r_1 = 4.77\text{mm}$, $r_2 = 7.79\text{mm}$, $r_3 = 11.44\text{mm}$, $r_4 = 7.54\text{mm}$, $w = 1.5\text{mm}$, $d_1 = 12.5\text{mm}$, $d_2 = 93\text{mm}$, $d_3 = 27\text{mm}$, $d_4 = 7\text{mm}$, $d_5 = 35\text{mm}$, $d_6 = 19.7\text{mm}$, $d_7 = 10\text{mm}$, $d_8 = 7\text{mm}$, $w = 0.7\text{mm}$, $d_{10} = 18.5\text{mm}$, $d_{11} = 2\text{mm}$, $h_1 = 42\text{mm}$, $h_2 = 10\text{mm}$, $h_3 = 0.5\text{mm}$, $h_4 = 7.5\text{mm}$, $h_5 = 8\text{mm}$, $h_6 = 4.5\text{mm}$, and $h_7 = 2.3\text{mm}$. The distances between the ground plan and center of the HB array RTDA antenna are 38mm and 83.3mm, respectively. It should be noted that the reason for different value of r_3 here (fabricated sample) with the simulated design in Fig. 3 of the SM is that the fabricated sample had 5% error in r_3 value with respect to the designed value.

Supplementary Note 4. Lowband performance: Cloaking surface radiator

To use the cloaking surface itself as a good radiator, it must support pure dipolar radiation without beam-squinting. Twisted double helix designs were first suggested in the conference paper [S10] to meet the large surface impedance needed; however, these covers suffered from narrow LB matching performance, beam squint, and cross-polarization. The simple design in this work has much better polarization purity giving squint-less dipolar radiation with excellent beamwidth. The main shortcoming of the design in Ref. [S10] was its similarity to a normal mode helix, which are inherently narrowband antennas [S11] .

In Fig. 4c of the main text, we have shown that the radiation patterns at the LB edges (699 and 969 MHz) match the conventional conductive dipole very well in terms of antenna gain, squint

and BW, and we will show even more comparisons later in this Supplementary Material. In Supplementary Fig. 5, we compare the matching performance measured from the two cross-dipoles in Supplementary Fig. 4e. The specified return loss of 10 dB is matched from 659-997 MHz, which exceeds the LB specification. The matching circuit was made for a single dipole, but when constructed into a cross-dipole, a narrowband spike at 914 MHz was observed with level -6.7 dB. While this spike is very narrow, it most likely due to near-field coupling between the two polarizations. Even still, the 10 dB LB bandwidth is 36%, which is quite good especially considering the low complexity integrated matching circuit and the HB transparency of the cross-dipole.

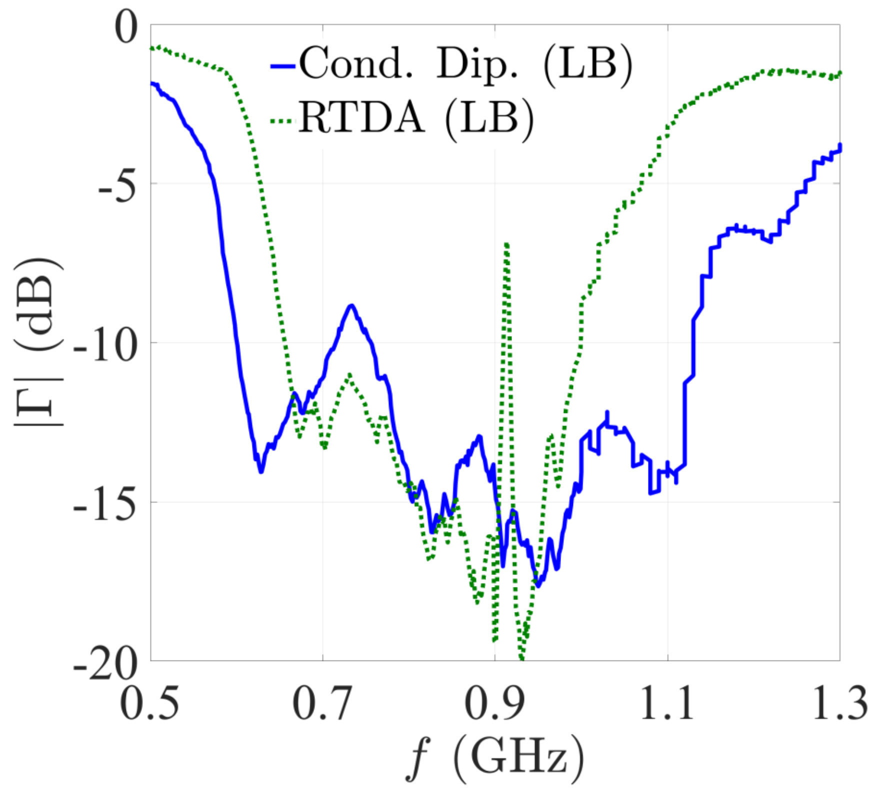

Supplementary Figure 5. Matching performance of the proposed radio-transparent dipole antenna based on a metasurface cloak (RTDA) and conventional conductive dipole (Cond. Dip.) lower band (LB) antennas.

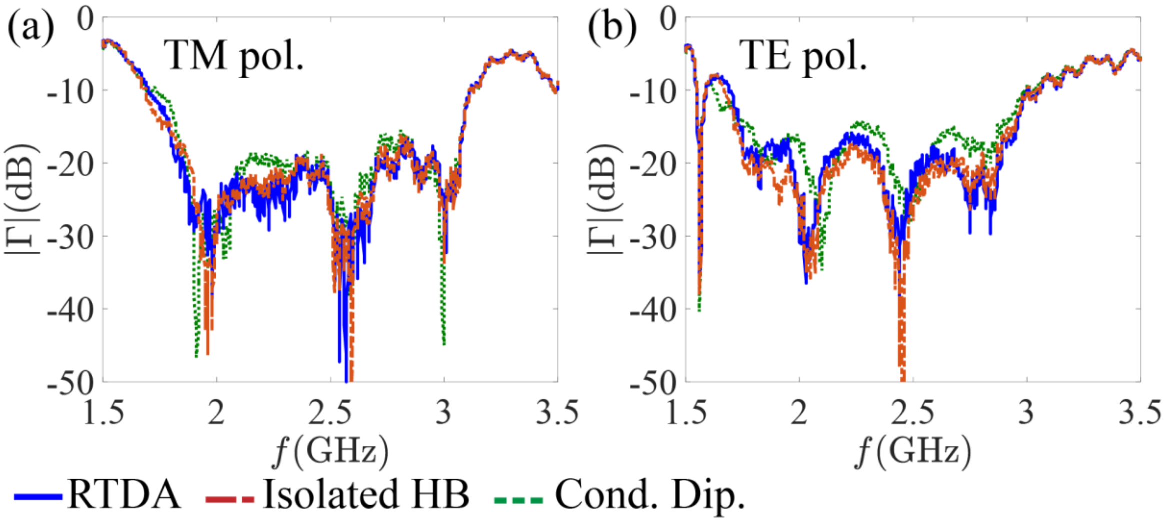

Supplementary Figure 6. Matching comparison between radio-transparent dipole antenna based on a metasurface cloak (RTDA), isolated higher band (HB), and conductive dipole (Cond. Dip.) antennas: (a) transverse electric (TE) and (b) transverse magnetic (TM) polarizations.

Supplementary Note 5. High-band matching performance

Supplementary Fig. 6 compares the matching of the HB elements without any LB element (Isolated HB), the RTDA, and the conductive dipole mounted in front of them. Here, we see both blockers have only a marginal effect on the matching of the HB elements. However, for the case of the TE polarization, some noticeable improvement is shown by the RTDA replacing the conductive dipole. We do note that for both polarizations, the RTDA does match the Isolated HB cases better across the HB.

Supplementary Note 6. Near-field verification for dominant TM polarization

It is interesting to consider the near-field effects of each of the LB antenna elements. Here we consider only the TM polarization because of its dominant scattering characteristics. The details of our in-house near-field scanner can be found in Ref. [S8] . For the following, the measurement raster scan area is taken over an area of 396×396 mm at a height of $z = 173\text{mm}$ (3/4 the conductive dipole length). The exciter in this case is a 5 dBi log-periodic antenna, which is the same antenna used as the receiver in the far-field measurements. Below in Supplementary Fig. 7, we plot the near-field scattering gain (SG) with background subtraction. This near-field figure of merit shows the difference in scattering between the RTDA and the conductive dipole. Remarkably, we see in-plane bistatic scattering suppression for over 3 GHz, most of which being below 6 dB. Compared to the cloak in Ref. [S3] , the increase in suppression bandwidth here is dramatic. We highlight five notable frequencies to compare the near-field snapshots-in-time in Supplementary Fig. 8 for impinging wavefronts polarized along $\mathbf{z}$ and propagating along $\mathbf{y}$.

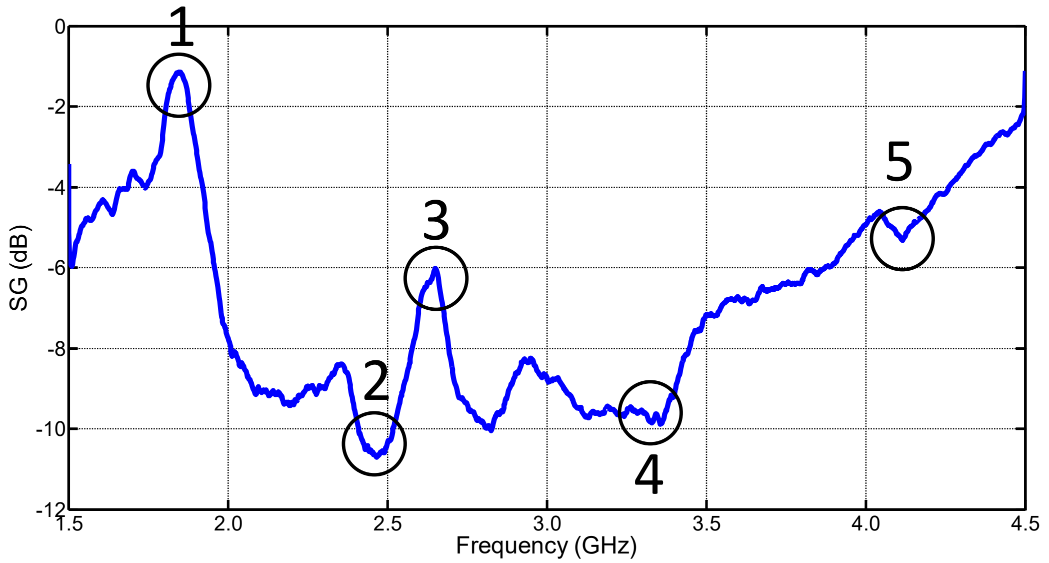

Supplementary Figure 7. Near-field scattering gain.

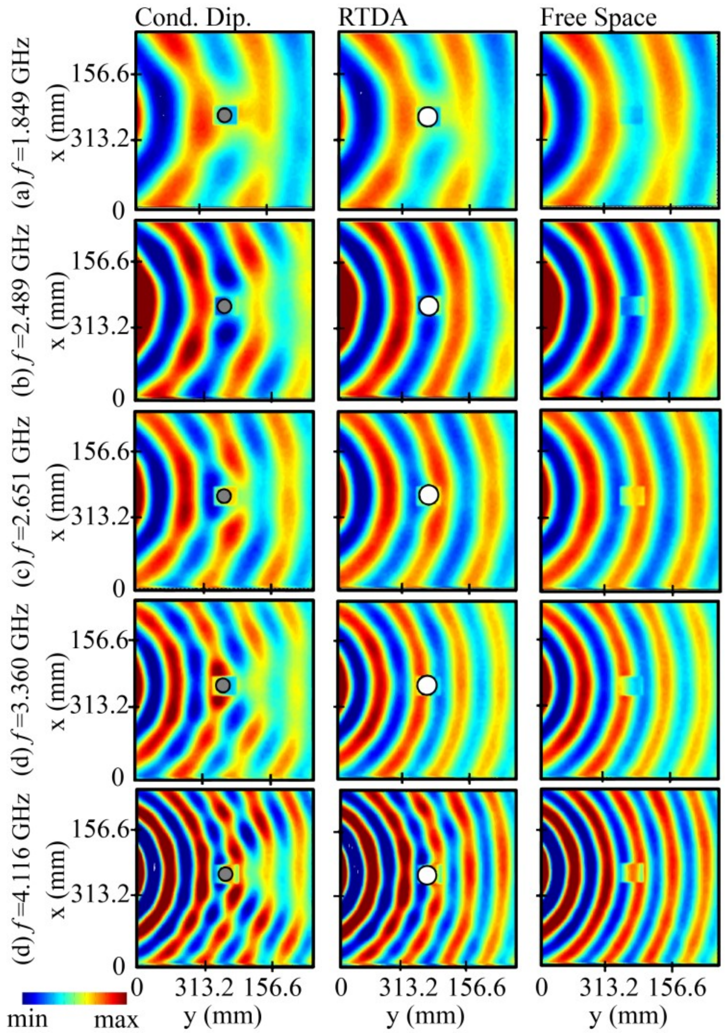

Supplementary Figure 8. Snapshots in time of the total electric field at different frequency points for conductive dipole, radio-transparent dipole antenna based on a metasurface cloak (RTDA), and free space scenarios (conductive dipole and RTDA cross-section to relative scale). All units are in mm.

Supplementary Note 7. Far-field measurement details

Supplementary Fig. 9 illustrates the setup of the far-field measurements of the main text. Here the unit cell is a 3×2 array of HB cross-dipoles, and the far-field measurements are compared by removing all LB elements (Isolated HB) and then mounting either the conductive dipole or RTDA, and taking measurements for all three cases. The receiver (Rx) in each of these measurements is a broadband log-periodic antenna with approximately 5 dBi gain across the bands of interest, and

the unit cell panel is the transmitter (Tx) in each case. The distance between the Rx and Tx is $R = 1.25\text{m}$ to be sufficiently in the far-field across the bands, according to $2l^2/\lambda$ [S11], where l is the longest dipole length and λ is the wavelength at the highest frequency.

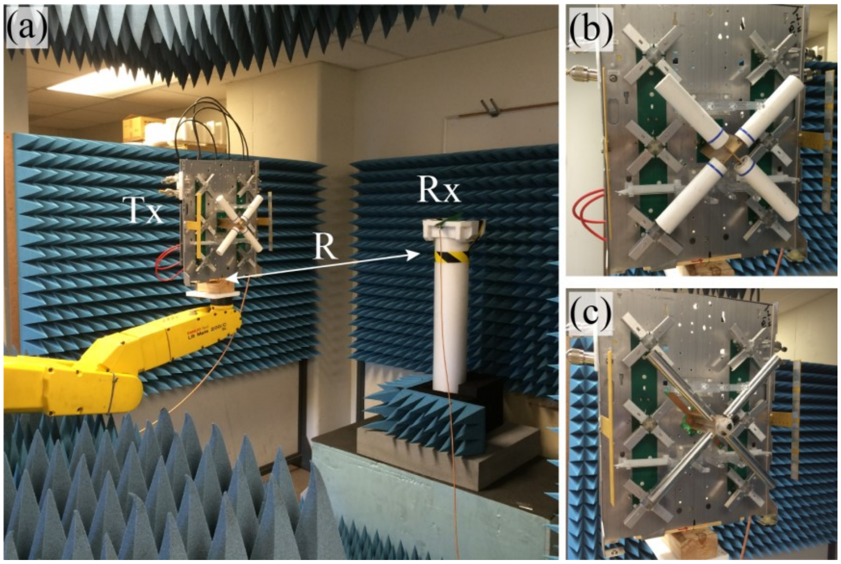

Supplementary Figure 9. (a) Antenna gain measurements for the low and high-bands. (b) Radio-transparent dipole antenna based on a metasurface cloak (RTDA) unit cell. (c) Conductive dipole unit cell.

To measure the gain in each scenario, we rotate the antenna panel using our robot, as seen in Supplementary Fig. 9a, and calculate $G_{Tx} = |S_{21}|^2 / \{G_{Rx}[\lambda/(4\pi R)]^2$, where S_{21} is the transmission measured from a vector network analyzer, and G_{Rx} is gain of the receiver [S11]. The gain of the receiver was first measured by using a pair of log-periodic antennas prior to the test shown in Supplementary Fig. 9a. In Supplementary Fig. 10, we show frequency samples not shown in the main paper to fully appreciate the HB antenna restoration by the RTDA approach.

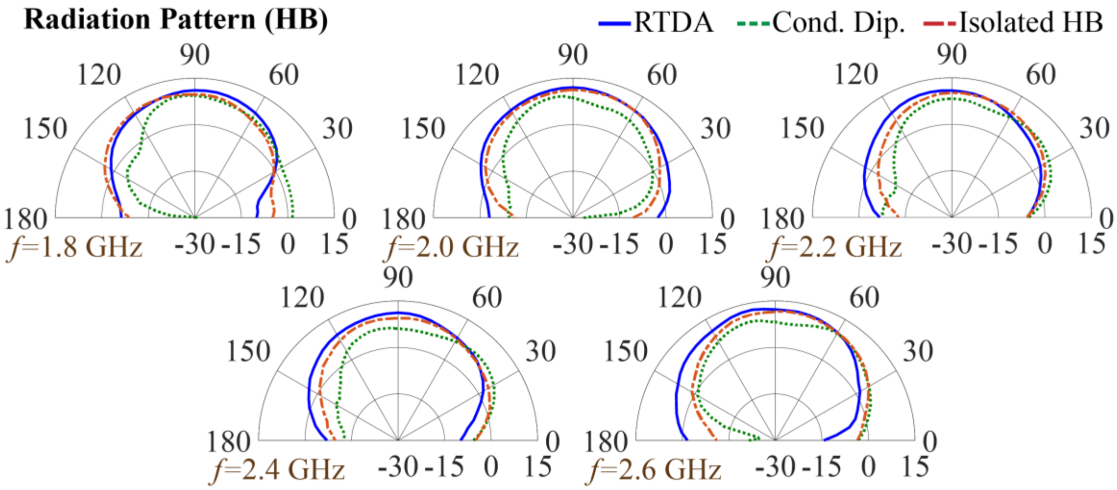

Supplementary Figure 10. Measured far-field results across higher band (HB) with each blocker, or of the exciter panel only. The results compare the performance of radio-transparent dipole antenna based on a metasurface cloak (RTDA), conductive dipole (Cond. Dip.), and Isolated HB cases.

Supplementary Note 8. Spectral and radiating response for a radio transparent Yagi-Uda antenna based on the proposed RTDA concept

To prove the universality of the proposed concept and the fact that the design is applicable to other types of antennas, we present in this section a radio transparent Yagi-Uda antenna based on the proposed RTDA concept. In contrast to a regular PEC antenna, this dielectric host-based antenna has a much reduced scattering cross section in the HB. The designed antenna is shown in the Supplementary Fig. 11. In this design, the length of the main dipole is 140mm and the reflector and director are 160 mm and 120 mm long, respectively. The dielectric host has $\varepsilon_r = 4.4$ and $\tan\delta = 0.0005$. Following the same design procedure that was explained throughout the paper, by the addition of an inductive metasurface, we are able to reduce the scattering of the host dielectric

while being able to used the metasurface for radiating purposes. The required surface impedance is $483.4j$ which is realized using the traces shown in Supplementary Fig. 11. The traces have a width of 1 mm. As expected, the designed radio transparent Yagi-Uda antenna radiates as a PEC Yagi-Uda antenna when fed and matched to a 50 Ohm port, while presenting and much reduced scattering cross section than the PEC Yagi-Uda antenna and the dielectric host at higher frequencies.

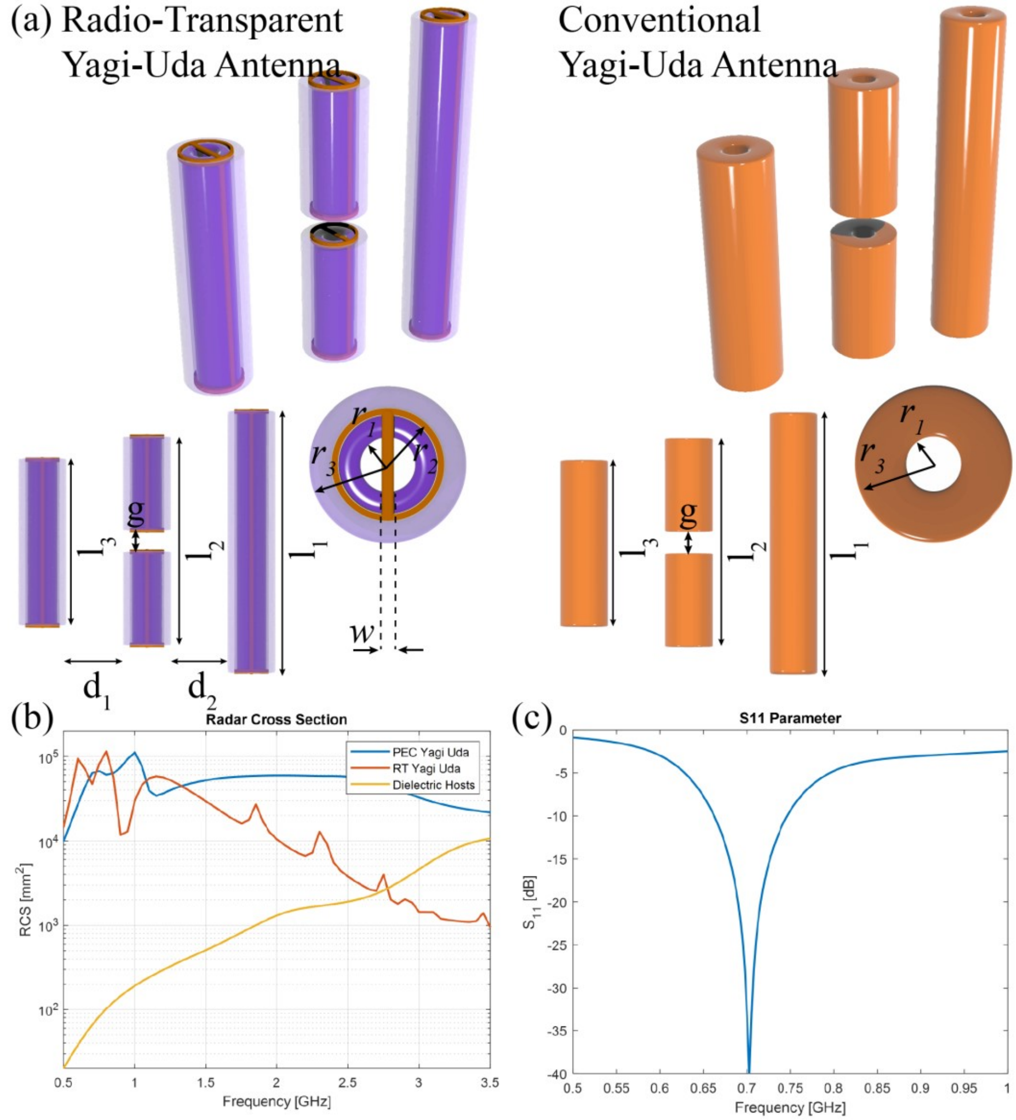

Supplementary Figure 11. (a) Geometry of the conventional and radio-transparent Yagi-Uda antennas under study (b) Spectral response for the designed radio-transparent dipole antenna based on a metasurface cloak (RTDA) based radio-transparent (RT) Yagi-Uda antenna. (c) S11 Parameter and radiation pattern for a RT Yagi-Uda antenna matched to a 50 Ohm Port. Simulations have been performed using CST STUDIO SUITE [S9] . Here, $l_1 = 160\,\text{mm}$, $l_2 = 140\,\text{mm}$, $l_3 = 120\,\text{mm}$, $r_1 = 7\,\text{mm}$, $r_2 = 8\,\text{mm}$, $r_3 = 10\,\text{mm}$, $w = 1\,\text{mm}$, and $g = 10\,\text{mm}$.

Supplementary References

[S1] A. Alù, "Mantle cloak: invisibility induced by a surface," *Phys. Rev. B*, vol. 80, 24115, 2009.

[S2] Z.H. Jiang, P.E. Sieber, L. Kang and D.H. Werner, "Restoring intrinsic properties of electromagnetic radiators using ultralightweight integrated metasurface cloaks", *Adv. Funct. Mater.*, vol. 25, no. 29, 4708−4716, June 2015.

[S3] J.C. Soric, A. Monti, A. Toscano, F. Bilotti, and A. Alù, "Dual-polarized reduction of dipole antenna blockage using mantle cloaks", *IEEE Trans. Antennas and Propagat.*, vol. 63, no. 11, 4827−4834, Sept. 2015.

[S4] A. Monti, J.C. Soric, A. Alù, F. Bilotti, A. Toscano, and L. Vegni, "Overcoming mutual blockage between neighboring dipole antennas using a low-profile patterned metasurface", *IEEE Antennas and Propaga. Lett.*, vol. 11, 1414−1417, Nov. 2012.

[S5] O. Luukkonen, C. Simovski, G. Granet, G. Goussetis, D. Lioubtchenko, A. V. Raisanen, and S. A. Tretyakov, "Simple and accurate analytical model of planar grids and high-impedance surfaces comprising metal strips or patches," *IEEE Trans. Antennas Propagat.*, vol. 56, pp. 1624−1632, 2008.

[S6] E.F. Knott, J.F. Shaeffer, and M.L. Tuley, *Radar Cross Section: its prediction, measurement, and reduction*, Artech House, MA, 1985.

[S7] B.A. Munk, *Frequency Selective Surfaces: Theory and Design*, Wiley, New York, 2000.

[S8] J.C. Soric, P.Y. Chen, A. Kerkhoff, D. Rainwater, K. Melin, and A. Alù, "Demonstration of an ultralow profile cloak for scattering suppression of a finite-length rod in free-space," *New J. Phys.*, vol. 15, 033037, March 2013.

[S9] CST STUDIO SUITE 2018, https://www.3ds.com/products-services/simulia/products/cst-studio-suite/.
[S10] J.C. Soric and A. Alù, "Radio-frequency transparent dipole antennas," *IEEE AP-S/URSI International Symposium*, 53, July 2015, Vancouver, BC.
[S11] C.A. Balanis, Antenna Theory: Analysis and Design 3rd Ed. Hoboken, NJ: Wiley, 2005.
